# De-ashed-biochar slow-release N fertilizer increased NUE in alkaline calcareous soils under wheat and maize crops

**DOI:** 10.1038/s41598-025-90651-7

**Published:** 2025-03-05

**Authors:** Muhammad Rashid, Qaiser Hussain, Khalid Saifullah Khan, Sarosh Alvi, Shokat Ali Abro, Muhammad Akmal, Shahzada Sohail Ijaz, Muhammad Umer, Abdul Ahad Qureshi, Mohamed S. Elshikh, Humaira Rizwana, Muhammad Rizwan, Rashid Iqbal

**Affiliations:** 1 Institute of Soil and Environmental Sciences, PMAS-Arid Agriculture University, Rawalpindi, 46300 Pakistan; 2 Soil Fertility Survey and Soil Testing Institute, Rawalpindi, 46300 Pakistan; 3 Department of Soil Sciences, Sindh Agricultural University, Tandojam, Pakistan; 4 Department of Biosciences, COMSATS University, Islamabad, Pakistan; 5 Department of Horticulture, PMAS-Arid Agriculture University, Rawalpindi, 46300 Pakistan; 6 Department of Botany and Microbiology, College of Science, King Saud University, Riyadh, 11451 Saudi Arabia; 7 Institute of Crop Science and Resource Conservation (INRES), University of Bonn, 53115 Bonn, Germany; 8 Department of Agronomy, Faculty of Agriculture and Environment, The Islamia University of Bahawalpur, Bahawalpur, 63100 Pakistan; 9 Department of Life Sciences, Western Caspian University, Baku, Azerbaijan

**Keywords:** Biochar-based fertilizers, N-dynamics, Nitrogen uptake, Nitrogen use efficiency, Agronomic benefits, Wheat, Maize, Plant sciences, Environmental sciences

## Abstract

Recently biochar has widely been reported as carrier of SRFs. However, the performance of SRFs synthesized from pristine biochar is still low and could not achieve the significant benefits compared to conventional N fertilizers. To overcome this limitation and research gap, BSRFs were synthesized using modified / de-ashed biochar as N-carrier. We hypothesized that BSRFs would NUE especially in alkaline calcareous soils for whom there is no specific SRF exist previously. In this study, the efficacy of BSRF formulated with 1:1 mass ratio of de-ashed biochar and urea was compared with CU and CSRF for improving NUE under wheat (*Triticum aestivum* L.) and maize (*Zea mays* L.) cropping system in two different textured soils. The results showed that compared to CU and CSRF, the addition of BSRF significantly increased the retention of soil mineral-N (NH_4_^+^-N, NO_3_^−^-N) which, consequently, enhanced the crops’ N-uptake up to 23.71% in wheat and 26.55% in maize. It was further observed that SOC contents were increased up to 50.79% in wheat and up to 47.61% in maize at harvest. The addition of BSRF enhanced the CEC up to 32.95% under wheat and up to 27.73% under maize, compared to CU. Eventually, BSRF significantly increased the grain yield and NUE of wheat by 12.04% and 40.44%, while the maize grain yield and NUE increased by 21.06% and 45.56%, respectively. This study concludes that BSRFs had a stronger yield-increasing effect than CU alone attributing to enhanced N retention and crop uptake in alkaline calcareous soils. It was also found that the de-ashed biochar is a strong candidate to formulate new SRFs with improved performance.

## Introduction

N availability is the most critical factor in alkaline calcareous soils, thereby limiting the optimum crop growth and productivity^1^. More than 70% of N applied through conventional fertilizers remains unavailable to crop plants because the conventional N fertilizers mineralized quickly in soil. As a result, plants are unable to absorb all released N. Therefore, more than half of N is easily lost through leaching^2^, volatilization^3^, and denitrification^4^. Due to these losses nitrogen-use-efficiency (NUE) is decreased significantly, especially in alkaline calcareous soils. In addition, the loss of N from soils, these losses pose economic and environmental concerns for society. Losses of N from agricultural fields often cause N deficiency and yield drag in crops with high N demand such as wheat and maize^5^.

Therefore, it is imperative to enhance the NUE in agricultural ecosystems, particularly in drylands to address challenges about food security, environmental degradation, and climate change^6^. Alkaline calcareous soils are characterized by poor organic matter (OM), high pH, and extreme temperatures. The widespread loss of SOM in alkaline calcareous soils is directly interconnected to poor NUE^7^. SOC which constitutes a significant proportion of SOM contributes to the retention of applied N. SOM provides numerous beneficial functions to crop production, including a high water holding capacity; the ability to provide, retain and recycle nutrients; and the capacity to buffer changes in pH, salinity and other chemical stressors^8^, suggesting that management practices that increase soil C can also reduce fertilizer N loss. There is, therefore, a strong reason to hypothesize that blending conventional mineral N fertilizers with organic materials such as composts, manures, lignite, and biochar as SRFs could reduce off-site fertilizer N loss since the organic materials occupy huge quantity of SOC / SOM^9^. Such SRFs will reduce the N losses and release the N for extended period of time to fulfill the crop needs by enhancing NUE.

Biochar (a carbonaceous material) poses many benefits as a soil amendment because it can improve N recycling in soil-plant systems^3^. Biochar has an extensive surface area, porosity, and a variety of functional groups making it capable of adsorbing various nutrient ions, such as ammonium, nitrate, phosphate, and potassium^10^. Recent studies have shown that BSRFs significantly increase the productivity of wheat, maize, rice, etc. while enhancing the total NUE^2,11,12^. However, the response of biochar is variable in acidic and alkaline soils^13^. Bhatnagar and Sillanpää^14^ indicated that the acid modification of biochar could increase the positive sites, which could facilitate to increase in the adsorption of anions^15^. It is well known that biochar significantly alters soil pH; however, its impact on calcareous soil is still unclear. The application of biochar to alkaline calcareous soils could have a negative impact due liming effect which could hinder the bioavailability of nutrients^16^. To overcome the alkalinity contributed by biochar, modification of biochar by deashing to remove the alkaline minerals/ ash could reduce the pH of biochar^17^. Additionally, higher recommended rates (20–40 t ha^-1^) of biochar as a soil ameliorant are uneconomical for its application on a large scale^9^. Therefore, it was hypothesized that de-ashed biochar as an N carrier to formulate BSRF is the more feasible and economical to counter N losses in alkaline calcareous soils to achieve sustainable crop production and to enhance NUE.

Wheat (*Triticum aestivum* L.) and maize (*Zea mays* L.) are the most important cereal crops in the world, especially in Pakistan and hence consume most fertilizers applications^13^. SRFs have the potential to ensure the nutrient supply during the entire crop growth period by a single dose, thereby saving the spreading costs and reducing the demand for manual labor required for top-dressing fertilizers^18^. Commercially available SRFs are costly which limits their use to only cash crops while only 1% of SRFs are used in large-scale crop production^19^. About 80 million tons of N fertilizers are applied to cereal crops every year worldwide and 50% of this N is consumed by wheat, maize, and rice^20^.

Keeping in view these research gaps and the significance of N fertilizers, it was hypothesized that integration of urea with de-ashed biochar as N-carrier could enhance the N retention in alkaline calcareous soils. Since, after de-ashing capacity of biochar to hold N was enhanced and its native pH was decreased. It was also hypothesized that BSRFs prepared using de-ashed biochar as carrier of N would improve the retention of soil mineral N in the soil profile and increase the NUE under two different crops i.e., wheat and maize in two different textured alkaline calcareous soils. We conducted two field experiments on wheat and maize as test crops on two different texture alkaline calcareous soils with objectives, (1) To evaluate the BSRF effects on the dynamics of soil mineral N, and (2) To assess the efficiency of BSRFs regarding crop N uptake, crop yield, and NUE. The experiments were conducted in two different soils to validated results under varying conditions.

## Materials and methods

### Preparation of BSRFs

The BSRFs were produced in this study at the Institute of Soil and Environmental Sciences, PMAS-Arid Agriculture University, Rawalpindi, Pakistan. Briefly, BSRFs were prepared by varying biochar-to-urea mass ratio of 1:1, 1:2, and 2:1. The urea solution was impregnated to de-ashed *Acacia arabica* L. biochar. Then, starch and PVA @ 10% of w/w basis was incorporated to urea impregnated biochar as adhesive to formulate pellets of BSRFs. XRD, FTIR, SEM characterization confirmed the effective interaction of urea to biochar. N release test in two different textured alkaline calcareous soils revealed that formulation of BSRF carrying 1:1 biochar to urea ratio was appropriate for crops like wheat and maize on the basis of pattern of N release. The detailed formulation procedure and characterizations of the BSRFs, conducted according to standards protocols, are reported in our article^21^. The composition of different N fertilizers used in this study is presented in Table 1.

**Table 1:** Characteristics of N fertilizers used in experiments.

Fertilizers	*N* contents (%)	pH	C (%)
CU	46.0	7.22	–
CSRF	26.0	6.90	–
BSRF	20.5	6.91	26

### Description of site

**Fig. 1:**
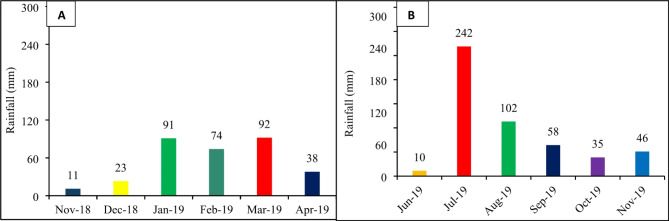
Rainfall distribution during (**A**) wheat and (**B**) maize season.

The study was conducted in two different textured alkaline calcareous soils, including (1) Gujranwala (fine-loamy, mixed, hyperthermic Typic, Haplustalf) and, (2) Pindorian (coarse-loamy, mixed, hyperthermic, Typic Haplustalf). Before execution of experiment, surface soil samples (0–20 cm) consisting of 3 sub-samples were collected from each location. The collected soil samples were homogenized separately, and then a subsample of was sieved (< 2 mm) and analyzed for baseline soil properties (Table 2). The climate of the area falls under a sub-humid, sub-tropical continental climate. The mean annual rainfall is about 584 mm, most of which falls during the monsoon season (mid-June to mid-September) and the remainder in winter (December to March). The mean annual temperature is 29.1˚C. Frost occurs during December and January [Reconnaissance Soil Survey, Campbellpur,^22^. The annual average rainfall data was obtained from local meteorological station (Fig. 1).

**Table 2:** The characteristics of soils used in experiments.

Soil characteristics	Unit	Coarse textured			Fine textured		
Soil particle size distribution	%	Sand	Silt	Clay	Sand	Silt	Clay
		53	30	17	19	54	27
Textural class	–	Sandy Loam			Silty clay loam		
pH	–	7.7			7.6		
EC	d Sm^− 1^	1.02			1.11		
OC	%	0.34			0.41		
Total-N	%	0.037			0.044		
NO_3_ˉ-N	mg kg^− 1^	7.4			8.1		
NH_4_^+^-N	mg kg^− 1^	6.3			7.4		
Available-P	mg kg^− 1^	4.7			5.4		
Extractable-K	mg kg^− 1^	92			94		
CaCO_3_	%	7.2			10.9		
CEC	cmol^+^kg^− 1^	11.3			13.1		

### Experimental set-up

The field experiments were conducted with 6 treatments, 2 soil types, and 3 replications for a total of 36 plots. Each plot was 4 m × 5 m, with a buffer zone of 0.8 m between plots and 1.0 m between blocks, arranged in a randomized complete block design (RCBD). The coordinates for site-1 were 33°55’55.4"N 72°27’27.6"E, and for site-2 were 33°56’06.4"N 72°26’49.4"E. The treatments used in this study were control (CK), conventional urea (U100) at 100% recommended dose of N, commercial slow-release fertilizer (C100) at 100% recommended dose of N, BSRF (B100) at 100% recommended dose of N, BSRF (B75) 75% recommended of N, and BSRF (B50) 50% recommended of N, respectively. The recommended NPK doses developed by Soil Fertility Research Institute, Punjab, Pakistan for wheat and maize were used in this study, viz., wheat 120, 100, 60 kg ha^− 1^, and maize 160, 120, 60 kg ha^− 1^. The whole recommended fertilizers were applied as a basal dose in both crops. Conventional urea (N 46%) and CSRF (N 26%) were purchased from Fatima Fertilizer Private (Ltd), Pakistan. The basic characteristics of soils are presented in Table 2. The recommended doses of N were applied as described in treatments while P and K were applied as SSP and SOP. The fertilizers were evenly applied in all plots and then incorporated into the top 0–20 cm soil. Generally used local cultivars of wheat (Galaxy) and maize (Pioneer 3025) were cultivated in the experiments. No extra irrigation was applied to wheat, whereas maize was irrigated as per requirement. All the management practices including hoeing, weeding, and pest management were kept the same as local farmers.

### Soil sampling and analysis

For the determination of soil mineral N (NH_4_^+^-N, NO_3_ˉ-N), soil samples were collected from (0–20 cm) periodically at 30, 60, and 90 days after sowing. At harvest, a composite soil sample (3 sub-samples) was collected from each plot to quantify the changes in pH, electrical conductivity (EC), soil organic carbon (SOC), and cation exchange capacity (CEC). The NO_3_-N and NH_4_-N contents were determined by extraction using 2 M KCl (soil: solution ratio, 1:10) followed by shaking for 1 h and later analyzing using the distillation method^23^. Soil pH and EC were determined by a 1:10 soil-to-water ratio using the pH and conductivity meter^24^. The SOC by 1 N K_2_Cr_2_O_7_ and H_2_SO_4_ extraction following Walkley and Black method is given by Nelson and Sommers^25^. The CEC was determined by 1 N sodium acetate solution, centrifuged at 3000 rpm and the supernatant was discarded four times. Then centrifugation was carried out with 33 mL ethanol. Sodium contents in the supernatant will be measured by a flame photometer (Digiflam-2000)^26^.

### Plant sampling and analysis

At maturity, wheat and maize were harvested from each plot. The biomass and grain yields were determined. Grains and biomass were dried in an oven at 60 °C for 24 h and powdered to determine the N-uptake, NAE, and NUE. Total N in plant samples was determined by following the Kjeldahl method, after digestion with concentrated H_2_SO_4_ and H_2_O_2_.

### Crop N-uptake

Nitrogen uptake by grain and biomass was calculated by multiplying grain and biomass yields with N concentration (%) in grain and biomass, respectively. The equations Eqs. (1), (2) were adopted to estimate the crop N-uptake.

1 $$N{\text{ }}uptake{\text{ }}by{\text{ }}grain{\text{ }}(kg{\text{ }}h{a^{ - \,1}}){\text{ }}={\text{ }}[N{\text{ }}content{\text{ }}in{\text{ }}grain{\text{ }}\left( \% \right) \times grain{\text{ }}yield{\text{ }}(kg{\text{ }}h{a^{ - \,1}})]/100$$

2 $$N{\text{ }}uptake{\text{ }}by{\text{ }}biomass{\text{ }}(kg{\text{ }}h{a^{ - \,1}}){\text{ }}={\text{ }}[N{\text{ }}content{\text{ }}in{\text{ }}biomass{\text{ }}\left( \% \right) \times straw{\text{ }}yield{\text{ }}(kg{\text{ }}h{a^{ - \,1}})]/100$$

### N agronomic efficiency and N use efficiency

The crop N contents and N-uptake in grain and biomass of wheat and maize were used to calculate nitrogen agronomic efficiency (NAE) and nitrogen use efficiency (NUE)^27^ by using the Eqs. (3), (4).

3 $$NAE{\text{ }}(kg{\text{ }}grain{\text{ }}k{g^{ - \,1}}N{\text{ }}applied){\text{ }}={\text{ }}[Grain{\text{ }}yield{\text{ }}(kg{\text{ }}h{a^{ - \,1}}){\text{ }}in{\text{ }}N{\text{ }}applied{\text{ }}plots--grain{\text{ }}yield{\text{ }}of{\text{ }}control{\text{ }}plots]/total{\text{ }}N{\text{ }}applied$$

4 $$NUE{\text{ }}\left( \% \right){\text{ }}={\text{ }}\left[ {\left( {N{\text{ }}uptake{\text{ }}by{\text{ }}the{\text{ }}fertilized{\text{ }}treatment - N{\text{ }}uptake{\text{ }}in{\text{ }}the{\text{ }}control} \right)/total{\text{ }}N{\text{ }}applied} \right] \times 100$$

### Statistical analysis

The data obtained was compiled and analyzed using Excel 2013. The soil and treatment differences were evaluated using a two-way analysis of variance (ANOVA), and the Tukey HSD test was performed for comparisons among treatments. All analyses were performed using the *p* ≤ 0.05 level.

## Results

### Effect of BSRF on soil chemical properties

**Fig. 2:**
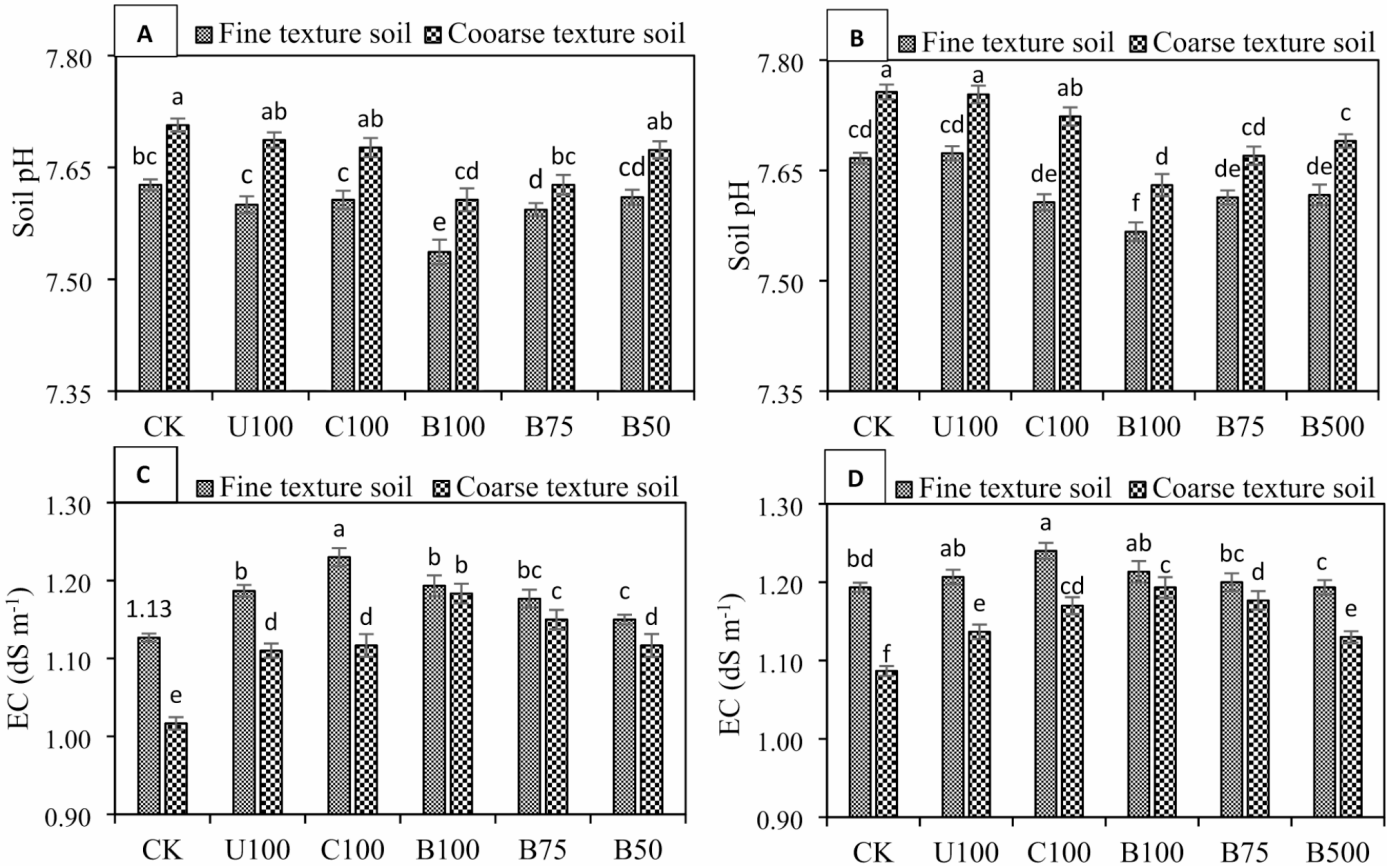
Effect of N fertilizers on soil pH in fine and coarse texture soils under (**A**) wheat (**B**) maize, and on soil EC in fine texture and coarse texture soils under (**C**) wheat (**D**) maize. Error bars represent the standard error of the mean of three replicates and different letters show significant differences among treatments at HSD *p* < 0.05. (CK = control, U100 = 100% recommended N by urea, C100 = 100% recommended N by CSRF, B100 = 100% recommended N by BSRF, B75 = 75% recommended N by BSRF, B50 = 50% recommended N by BSRF).

**Fig. 3:**
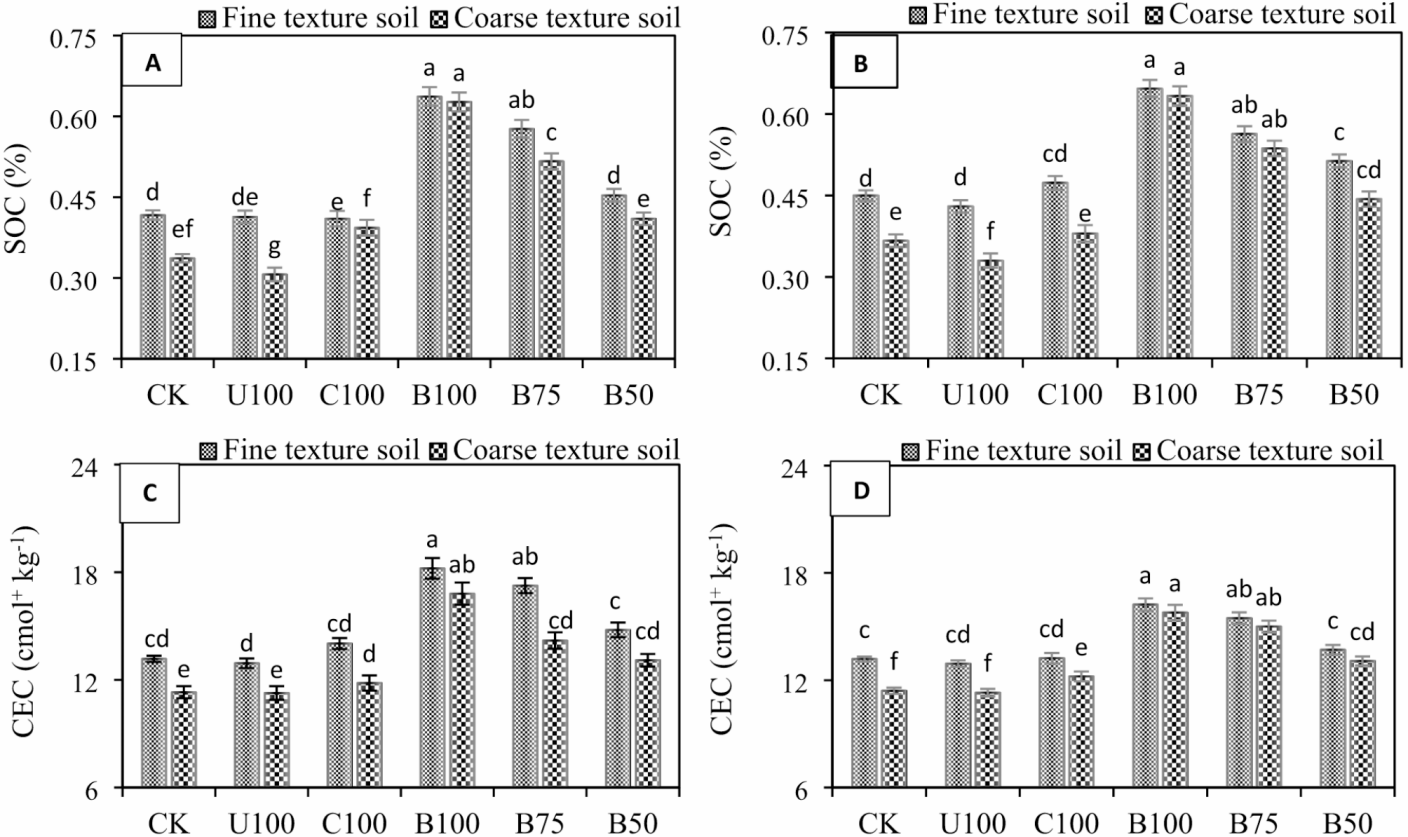
Effect of N fertilizers on SOC contents in fine and coarse texture soils under (**A**) wheat (**B**) maize, and on soil CEC in fine texture and coarse texture soils under (**C**) wheat (**D**) maize. Error bars represent the standard error of the mean of three replicates and different letters show significant differences among treatments at HSD *p* < 0.05. (CK = control, U100 = 100% recommended N by urea, C100 = 100% recommended N by CSRF, B100 = 100% recommended N by BSRF, B75 = 75% recommended N by BSRF, B50 = 50% recommended N by BSRF).

The addition of all N fertilizers significantly (*P* ≤ 0.05) decreased the soil pH in both wheat and maize crops under both soils, compared to the control (Fig. 2A–D). However, the decrease in soil pH was more evident in BSRFs treated soils compared to CU and CSRF. In wheat, the lowest soil pH (7.54) and (7.61) was observed in BSRF-amended soils with 100% recommended N dose in fine and coarse texture soils, respectively. Soil pH, on the other hand, in CU-treated soil was 7.60 and 7.69, respectively, in fine and coarse-texture soils. Similarly, in maize, the lowest soil pH (7.57 and 7.63) was recorded in BSRF-amended soil with 100% recommended N dose in coarse and fine texture soils, respectively, compared to 7.67 and 7.76 in CU treated soils. Soil EC was also influenced by all N fertilizers and an increase in EC was noted with the addition of N fertilizers. The maximum increase in soil EC in fine and coarse texture soils under both wheat and maize was observed with CSRF followed by BSRF-amended soils with 100% recommended N dose which was statistically at par with CU treatment. The results showed that application BSRFs significantly (*P* ≤ 0.05) increased SOC compared to CK, CU, and CSRF treatments both in wheat and maize under fine and coarse texture soils (Fig. 3A, B). The highest SOC contents 0.64% and 0.63% were recorded in BSRF-amended soils with 100% N dose in fine and coarse texture soil, respectively, while the lowest SOC contents (0.41% and 31%) were observed for CU treatment in fine and coarse texture soils, respectively, under wheat crop. Similarly, the highest SOC (0.65% and 0.63%) in fine and coarse texture soils, respectively, under maize crop were also noted for BSRF-amended soils with 100% recommended N dose. The results also showed that introduction of BSRFS significantly (*P* ≤ 0.05) improve the soil CEC in both soils under both crops (Fig. 3C, D). BSRFs increased the soil CEC by 27.67% and 32.95%, respectively in fine and coarse texture soils under wheat and 18.73% and 27.73% in fine and coarse texture soils under maize crop, compared to CK.

### Effect of BSRFs on soil mineral-N retention

**Fig. 4:**
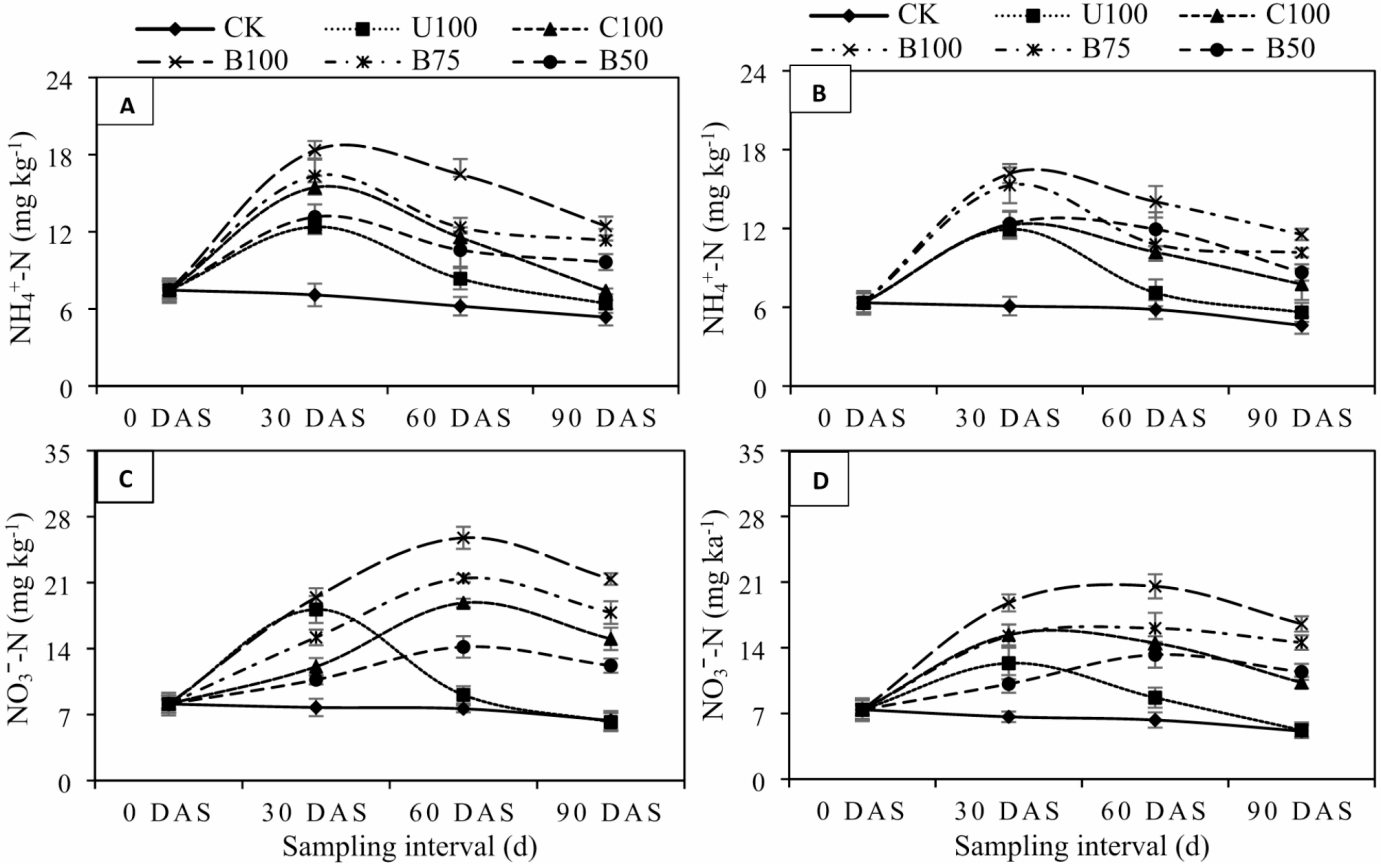
Effect of N fertilizers on (**A**) NH_4_^+^-N in fine texture (**B**) NH_4_^+^-N in coarse texture (**C**) NO_3_ˉ-N in fine texture (**D**) NO_3_ˉ-N in coarse texture soil under wheat. Error bars represent the standard error of the mean of three replicates. (CK = control, U100 = 100% recommended N by urea, C100 = 100% recommended N by CSRF, B100 = 100% recommended N by BSRF, B75 = 75% recommended N by BSRF, B50 = 50% recommended N by BSRF).

**Fig. 5:**
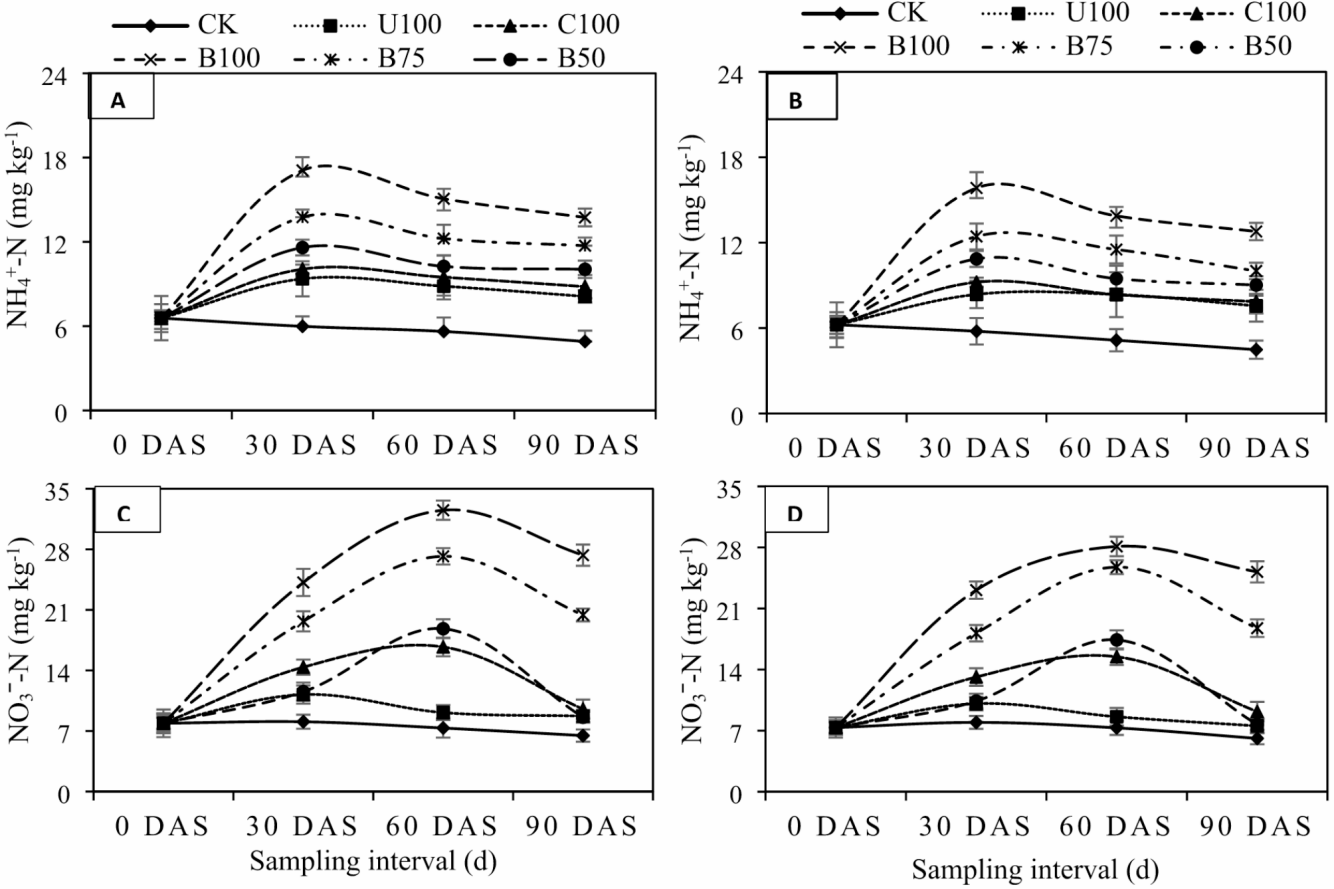
Effect of N fertilizers on (**A**) NH_4_^+^-N in fine texture (**B**) NH_4_^+^-N in coarse texture (**C**) NO_3_ˉ-N in fine texture (**D**) NO_3_ˉ-N in coarse texture soil under maize. Error bars represent the standard error of the mean of three replicates. (CK = control, U100 = 100% recommended N by urea, C100 = 100% recommended N by CSRF, B100 = 100% recommended N by BSRF, B75 = 75% recommended N by BSRF, B50 = 50% recommended N by BSRF).

The results showed significantly (*P* ≤ 0.05) higher concentrations of mineral N (NH_4_^+^-N, NO_3_ˉ-N) in soils fertilized with CU, CSRF, and BSRFs compared to CK (Figs. 4 and 5-A–D). However, application of BSRFs compared to CU and CSRF enhanced significantly higher quantity of mineral N in both soils under both crops. In both soils under both crops, NH_4_^+^-N concentrations decreased with time, however, this decrease was significantly faster in CU and CSRF treatments while it was quite slow and gradual in BSRFs treatments. Similarly, soil NO_3_ˉ-N concentrations first increased till the 60th day after application of N fertilizers and then decreased significantly in all treatments. But, after 90 days of fertilizer application, it was noticed that BSRFs treatments still maintained the critical NO_3_-N contents (> 20 mg kg^− 1^) required for optimum crop growth compared to CU and CSRF which hardly maintain the critical NO_3_ˉ-N concentrations till the 60th day of fertilizer application. After 30 days of fertilizer application, compared to CU, on average, the incorporation of BSRFs increased the NH_4_^+^-N contents by 32.49% in fine-texture soil and 26.21% in coarse-texture soil under wheat. In maize, the addition of BSRF increased the NH_4_^+^-N contents by 45.20% in fine-texture soil and 47.22% in coarse-texture soil. Similarly, BSRFs significantly increased the NO_3_ˉ-N contents by 6.63% in fine texture soil and 34.27% in coarse texture soil under wheat while, 53.71% in fine texture soil and 56.32% in coarse texture soil under maize, compared to CU (Fig. 4A, B, C, D). It was also observed that BSRFs retained more mineral N in coarse-texture soils compared to fine-texture soils under both crops.

### Effect of BSRFs on wheat and maize grain and biomass yields

**Fig. 6:**
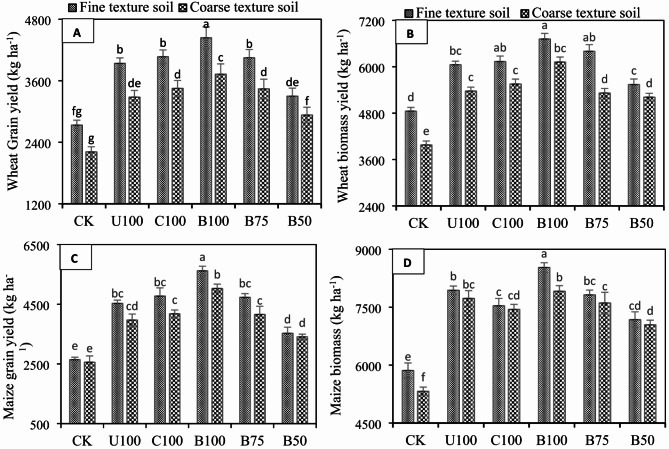
Effect of N fertilizers on (**A**) wheat grain yield (**B**) wheat biomass yield (**C**) maize grain yield (**D**) maize biomass yield in fine texture and coarse-texture soils. Error bars represent the standard error of the mean of three replicates and different letters show significant differences among treatments at HSD *p* < 0.05. (CK = control, U100 = 100% recommended N by conventional urea, C100 = 100% recommended N by CSRF, B100 = 100% recommended N by BSRF, B75 = 75% recommended N by BSRF, B50 = 50% recommended N by BSRF).

The addition of all N fertilizers irrespective of source significantly (*p* ≤ 0.05) increased the wheat and maize grain and biomass yields under both soil types (Fig. 6A–D). However, compared to CU, application of BSRF significantly increased the wheat grain yield up to 11.24% in fine texture soil, and 12.01% in coarse-texture soil whereas the application of BSRF significantly increased the wheat biomass yield up to 9.90% in fine texture soil and 12.24% in coarse texture soil, compared to CU. Similarly, addition BSRF significantly increased the maize grain yield up to 19.40% in fine-texture soil and 21.06% in coarse-texture soil, compared to CU. The same trend was observed in maize biomass yield and it was observed that BSRF significantly increased the maize biomass yield up to 6.91% in fine-texture soil and up to 2.35% in coarse-texture soil, compared to CU. The results also showed that the benefits of BSRF were more prominent in coarse-texture soil compared to fine-texture soil.

### Effect of BSRFs on crop N-uptake

**Fig. 7:**
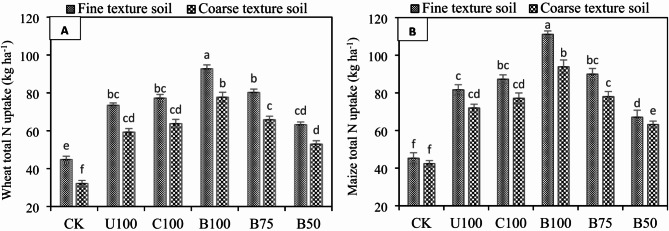
Effect of N fertilizers on (**A**) wheat total N uptake (**B**) maize total N uptake in fine texture soil and coarse-texture soil. Error bars represent the standard error of the mean of three replicates and different letters show significant differences among treatments at HSD *p* < 0.05. (CK = control, U100 = 100% recommended N by urea, C100 = 100% recommended N by CSRF, B100 = 100% recommended N by BSRF, B75 = 75% recommended N by BSRF, B50 = 50% recommended N by BSRF).

The data showed that BSRF significantly (*p* ≤ 0.05) enhanced the N uptake in both crops under both soils, compared to CU (Fig. 7A, B). In wheat, BSRF significantly enhanced the crop N uptake up to 20.80% in fine texture soil and 23.71% in coarse-texture soil, respectively, compared to CU. Similarly in maize, BSRF significantly increased the N uptake up to 26.55% in fine-texture soil and up to 23.32% in coarse-texture soil, compared to CU.

### Effect of BSRF on NAE and NUE

**Fig. 8:**
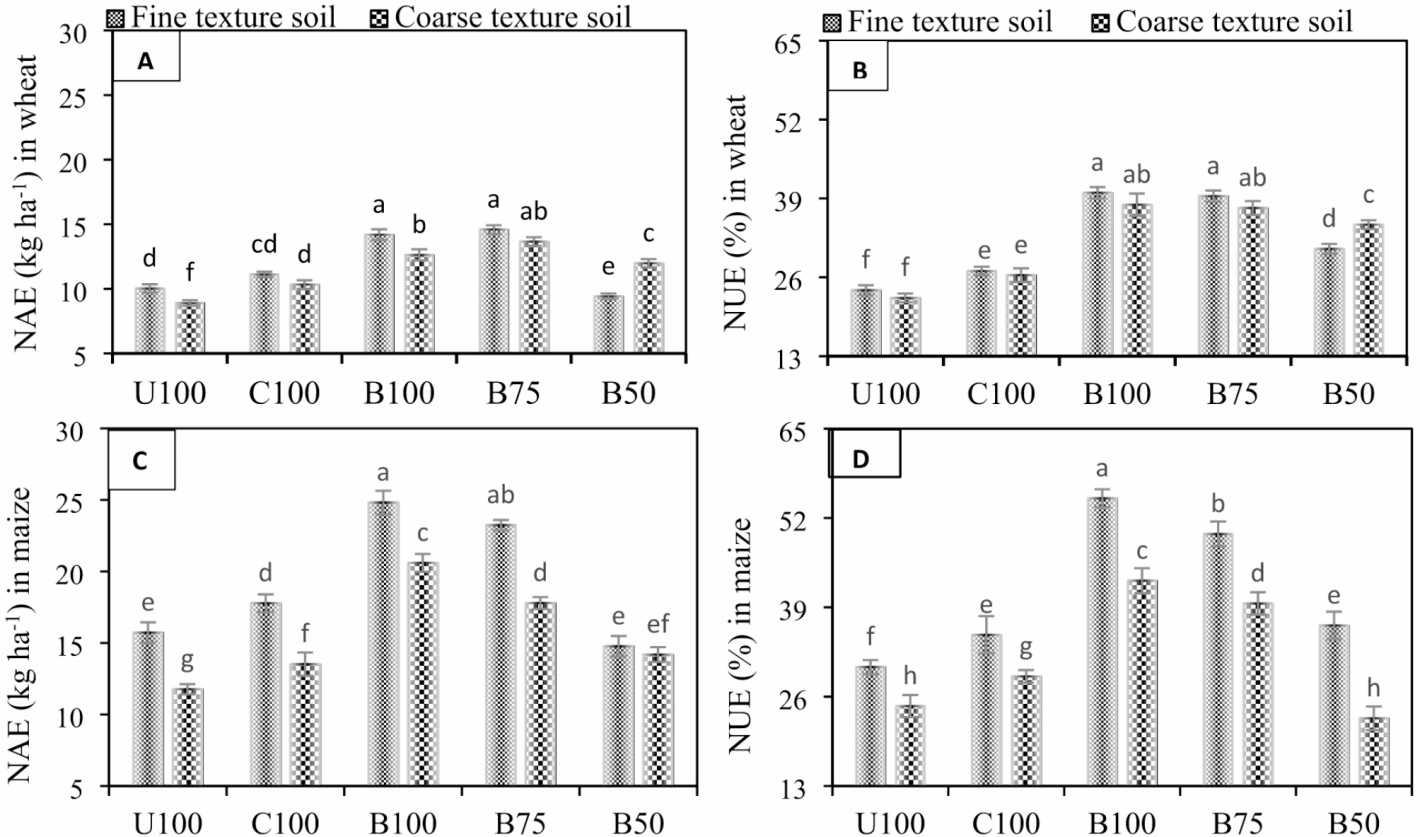
Effect of N fertilizers on (**A**) NAE in wheat (**B**) NAE in maize (**C**) NUE in wheat (**D**) NUE in maize in fine texture and coarse-texture soils. Error bars represent the standard error of the mean of three replicates and different letters show significant differences among treatments at HSD *p* < 0.05. (U100 = 100% recommended N by urea, C100 = 100% recommended N by CSRF, B100 = 100% recommended N by BSRF, B75 = 75% recommended N by BSRF, B50 = 50% recommended N by BSRF).

Nitrogen agronomic efficiency (NAE) and nitrogen use efficiency (NUE) were affected significantly (*p* ≤ 0.05) by the addition of different N fertilizers (Fig. 8A, B, C, D), compared to CK. It is worth mentioning that BSRFs significantly improved the NAE up to 31.25% and 34.85% while NUE up to 40.24% and 40.44% in fine and coarse texture soils, respectively, compared to CU. Similarly, in maize, application of BSRFs significantly enhanced the NAE up to 36.62% and 42.86% while NUE 44.76% and 42.56% in fine and coarse texture soils, respectively, compared to CU.

## Discussion

For manufacturing SRFs, the scientists have given top priority to feedstock and coating materials. Because, the properties of nutrient carriers viz., surface area, porosity, pore volume, and functional groups as well as the mixing, encapsulation, or granulation qualities determine the quality and performance of SRFs^28^. Although biochar is the best natural nutrient carrier^29^, however in addition to biochar, starch^30^ and rice husk^31^ were also found effective. We chose acacia wood biochar to produce BSRFs because it is rough and porous, with visible cracks in the inner layer^32^. Its porous nature facilitates the entry of urea and water which ultimately leads to degradation owing to water uptake^2,29^. BSRFs can improve soil physicochemical properties^21^, extend N-retention^33^, crop yield^34^, and NUE^35^. It is well-established that biochar is an organic amendment having an aromatic structure that can be retained in soils for centuries (Farrell et al. 2014). Biochar can hold higher amounts of essential plant nutrients due to its high surface area, porosity, CEC, and surface functional groups^36^. In this study, BSRFs were evaluated to assess their performance in alkaline calcareous soils. Earlier, no specific BSRF was synthesized for these soils. The research data regarding improving the NUE in alkaline calcareous soils are either limited or unavailable. These soils are confronted with severe issues like poor nutrient and water retention and NUE. Therefore, these soils require special research attention as they occupy one-third of the cultivated lands. High soil pH, low SOM, and calcareousness are primary challenges/obstacles regarding improving the efficiency of applied fertilizer nutrients. This study, therefore, preliminary attempt to test designer slow-release fertilizer to combat the challenges of these soils.

### Effect of BSRFs on soil chemical properties

In this study, BSRF significantly (*P* ≤ 0.05) changed the chemical properties (pH, EC, SOC, and CEC) of two different textured alkaline calcareous soils. Soil pH substantially impacts the nutrient availability and mobility in the root zone^37^. In the present study, at harvest of both crops BSRFs significantly decreased the pH of both soils. The decrease in soil pH is attributed to the excessive release of protons (H^+^) from the exchange sites of de-ashed biochar from BSRFs. In addition, the release of organic acids during the decomposition of organic matter present in the soil might also contribute to reduce soil pH^38^. It has extensively been reported that the addition of biochar to acidic soils increases the soil pH due to its liming effect^39^. However, soil pH decreased after the application of BSRFs because de-ashing leached the alkaline components of biochar and enhanced the carboxylic functional groups which when used as N carrier reduced the soil pH. Sahin et al.^17^ confirmed that only acidified biochars are effective in reducing soil pH under calcareous conditions. Soil EC is mainly dependent on ash contents of biochar. De-ashing removed the indigenous ash minerals from biochar, therefore, the addition of BSRFs to the soil decreased the soil EC significantly. Non-acidified / pristine biochar due to higher indigenous alkalinity increased the soil pH and EC for a short time^40^. This study was conducted in alkaline calcareous soils where increasing soil pH and EC was not desirable. Therefore, de-ashed biochar as N carrier was more suitable for alkaline calcareous soils. The addition of BSRFs significantly increased the soil CEC compared to CU and CSRF. The increase in CEC was attributed to large surface area and porosity of biochar in BSRF. Previously, it was studied that a dramatic increase in the CEC of biochar is observed after deashing and ozonization^41^. Oxygen-containing surface functional groups are responsible for higher CEC in low-temperature biochar^42^. Although CEC is a natural and inherent characteristic of any soil, management does not easily change it. However, many studies have shown that charred materials like biochar due to the presence of strong carboxylic and phenolic functional groups with a negative charge on the surface can promote soil CEC^43^. In alkaline calcareous soils, high soil pH and atmospheric temperature are responsible for the rapid mineralization of SOM. However, during this study we found that BSRFs significantly increased the SOC in both soils after harvest of wheat and maize. This increase was due to stability of biochar in soil which regulated the SOC in soil by promoting the soil microbial activity^44^. Biochar in soil is very stable against decomposition^45^. The storage of SOC also increases soil C sequestration and helps in sustainable crop production^46^. Despite the fact, that a precise amount of biochar is added to the soil through BSRFs, still a significant increase in SOC has been reported in the literature. Zheng et al.^47^ found that biochar-based nitrogen fertilizers (BBNFs) when applied at 225 kg ha^− 1^ resulted in a dramatic increase of 16% in SOC compared to conventional N fertilizers. Similarly, Liao et al.^4^ revealed that SOC was increased by 20% by BBNFs compared to urea. It was also assumed that the addition of de-ashed biochar into alkaline soil as an N carrier could have ameliorated the soil alkalinity and could have enhanced the C sequestration. Due to C sequestration by BSRFs, carbon dioxide discharge may have reduced and improved the SOC contents in alkaline calcareous soils^13^.

### Effect of BSRFs on soil mineral-N retention

The application of BBNFs have been considered as the most efficient strategy for extended N retention in the soil thereby, enhancing the NUE^48,49^. In this study application of BSRFs significantly enhanced soil mineral N retention. It was observed that the concentration of NH_4_-N was increased significantly in all treatments after the application of all N fertilizers compared with the CK. However, the increase in NH_4_^+^-N was much higher in BSRFs treatments compared to CU. The NO_3_ˉ-N concentrations, on the other hand, increased the after application of N fertilizers, and then decreased. However, NO_3_ˉ-N decreased moderately in BSRFs while decreasing rapidly in CU, confirming the slow release of N from BSRFs. This slower release was likely due to the delayed hydrolysis of N from the BSRFs^50^. Since, the extensive surface area, porosity, CEC, and reactive surfaces contribute to the adsorptive nature of biochar, which has been exploited to synthesize BBNFs^49^. By providing this control, the large mineral N pools that can occur following mineral fertilizer application are avoided, thus minimizing N loss pathways including denitrification^35^, leaching^51^, and volatilization^52^. Dong et al.^48^ reported that NH_4_^+^-N concentration could be reduced by 55% in percolation water after replacing compound fertilizers with BBNF. The conversion of NH_4_^+^-N to NO_3_ˉ-N is an oxidation process that releases protons in the soil causing a decline in soil pH that confirms the process of nitrification^53^. The continuous release of N from BSRFs increased soil NO_3_ˉ-N contents in the later crop growth stages^54^. Wang et al.^55^ also observed that NO_3_^−^ leaching loss was the predominant pathway of N loss and contributed to 52–96% of total N leached from BBNFs. But compared with urea and NH_4_NO_3_, NO_3_^−^ leaching loss was reduced by over 60% with BBNFs^56^. In addition, de-ashed (acidified) biochar proved effective in decreasing soil pH and increasing the SOC. This decrease in soil pH and increase in SOC might be the main reasons for improving N retention by decreasing the NH_3_ losses in BSRFs treatments.

### Effect of BSRFs on wheat and maize grain and biomass yields

Biochar used ingredient of BSRFs which enhanced the wheat and maize biomass and grain yield compared to CU. We observed that application of BSRF even at reduced rate (75% of recommended N) for wheat and maize yielded statistically similar crop yields compared to CU and CSRF. Therefore, BSRFs has ability to reduce the recommended rates of N in alkaline calcareous soils up to 25%. It was also noticed that wheat and maize yields were higher in fine texture soil but the benefits of BSRFs were more evident in coarse texture soil compared to fine texture soil. Biochar improves crop yields in coarse-textured soils more than in fine-textured soils because it improves the soil’s physical and chemical properties. This includes increasing the soil’s water retention and nutrient availability. It might be due to improved N utilization due to the extended N bioavailability after the application of BSRFs. Because it is a fact that an ample supply of N is crucial to maintain a healthy crop stand. The appropriate synchrony between N addition and crop needs was improved by BSRFs which, consequently, increased the biomass and grain production. In addition to this, enhanced SOC and CEC after BSRFs could have ensured a better supply of other required nutrients as well. Several studies carried out in different agricultural systems also reported that the application of BBNFs enhanced the N availability which increased the dry matter yield and N uptake^50,52^. BSRFs performed better in coarse texture soil.

### Crop N-uptake

The addition of biochar to soil could substantially enhance the retention of plant-available nutrients. The present study showed that BSRFs addition significantly increased NO_3_-N and NH_4_-N concentrations in different soils. Crop N uptake is determined by soil available N content, as BSRFs significantly increased the contents of NO_3_-N and NH_4_-N in both soils under both crops, which increased the N uptake significantly in this study. In addition, BSRFs had better potential to maintain better soil moisture which could have also enhanced the N uptake, as the shortage of soil moisture, especially during the wheat growing season is a limiting factor in alkaline calcareous soils. Gao et al.^49^ investigated that BBNFs ensure the supply of available N during the late growth stages of crops to support growth, thereby promoting N uptake by improving the physical conditions of the soil and greater water availability enhanced nutrient-absorption capacity of the crop. Similar findings were presented by Khan et al.^57^ that the capacity of biochar to absorb N reduces the N toxicity in the early stages of plant growth and then enhances the N availability in the middle and late stages of plant growth to meet the plant N requirements. Furthermore, the biochar also provides N, P, and K to the soil in bioavailable forms, which might have promoted plants’ nutrient supply and uptake to elevate the rapeseed growth and yield^58^.

### Effect of BSRF on NAE and NUE

BSRFs significantly enhanced the NAE and NUE of wheat and maize under both soils. The lower NUE of conventional N fertilizers not only results in low crop yield but also increases the production cost. Therefore, improving the efficiency of fertilizers is of utmost importance for developing sustainable agriculture. In various studies it has been proposed that carbon-poor, less fertile alkaline calcareous soils have been the potential hotspots where biochar amendments may be used to enhance carbon sequestration and provide additional soil fertility benefits^59^. Lu et al.^60^ described that the reduced N application may affect severely the plant growth and grain yield. A conclusion presented by Ladha et al.^61^ suggested that the appropriate management of N application is compulsory to increase its N uptake. On the other hand, present-day cultivars of wheat and maize require a higher N input which contrarily causes a risk of environmental pollution and increases production costs. N uptake and efficiency is the measure of how much N is taken up by the crop plants. To ensure ample N uptake the N source as fertilizer should regulate the soil N supply to increase NUE^62^. However, N fertilization should be matched with the crop demand as, in our case, the higher agronomic yield was achieved by the new N formulations. Hawkesford et al.^63^ studied that only 33% of the applied N fertilizer is recovered in the harvested grains while a huge quantity of 67% of N is lost, which is also a major source of pollution. We found that net benefits of BSRFs including NUE were substantially higher in coarse texture soil than fine texture soil. Because the water and nutrient holding capacity of coarse texture soil was initially very low due low CEC and clay content compared to fine texture soil. BSRFs containing biochar as ingredient improved the soil structure and CEC which improved the growth conditions^64^.

## Conclusion

The results of our study demonstrate the significant impact of BSRFs on alkaline calcareous soils. Previously, specified SRFs for these soils was an unexplored area of research. The findings reveal that the intervention of BSRFs significantly improved the soil chemical properties, including reduced soil pH and improved SOC and CEC. Moreover, BSRFs ensured the consistent supply of N throughout the wheat and maize growth cycles, leading to increased crop N uptake and grain yields compared to CU due to which NUE enhanced drastically. It was concluded that de-ashed biochar is very effective in producing targeted BSRFs for alkaline calcareous soils, where N losses are markedly high. Furthermore, de-ashed biochar may offer economic benefits by reducing the required quantity of biochar as an N carrier due its higher ability to hold more N. This study provides valuable insights of several avenues for future research. During this study we focused on chemical modification of biochar with dilute HCl. However, investigating other modification methods of biochar and variable compositions of BSRFs could lead to the development of more efficient BSRFs. In addition, assessing the potential benefits of BSRFs in non-calcareous soils could expand their applicability and impact. We believe that conducting life cycle assessments and cost-benefit analysis would provide a more comprehensive understanding of BSRFs’ viability. By addressing these knowledge gaps, future research can further optimize BSRFs and unlock their potential to improve soil fertility, crop productivity, and environmental sustainability.

## Data Availability

All the raw data in this research can be obtained from the corresponding authors upon reasonable request.

## Abbreviations

ANOVA: Analysis of variance
BBNFs: Biochar-based nitrogen fertilizers
BSRF: Biochar based slow-release fertilizer
C: Carbon
CEC: Cation exchange capacity
CSRF: Commercial slow-release fertilizer
CU: Conventional urea
EC: Electrical conductivity
HSD: Honest significant test
K: Potassium
N: Nitrogen
NAE: Nitrogen agronomic efficiency
NUE: Nitrogen use efficiency
OM: Organic matter
P: Phosphorus
PMAS: Pir Mehr Ali Shah
RCBD: Randomized complete block design
SOC: Soil organic carbon
SOP: Sulfate of potash
SRFs: Slow-release fertilizers
SSP: Single super phosphate

## References

[1] Umar, W., Czinkota, I., Gulyás, M., Aziz, T. & Hameed, M. K. Development and characterization of slow release N and Zn fertilizer by coating Urea with Zn fortified nano-bentonite and ZnO NPs using various binders. *Environ. Technol. Innov.* **26**, 102250 (2022).

[2] Liu, M. et al. Nitrogen leaching greatly impacts bacterial community and denitrifiers abundance in subsoil under long-term fertilization. *Agric. Ecosyst. Environ.* **294**, 106885 (2020).

[3] Sun, H. et al. Wheat straw Biochar application increases ammonia volatilization from an urban compacted soil giving a short-term reduction in fertilizer nitrogen use efficiency. *J. Soils Sediments*. **19**, 1624–1631 (2019).

[4] Liao, J. et al. Effects of biochar-based controlled release nitrogen fertilizer on nitrogen-use efficiency of oilseed rape (*Brassica Napus* L). *Sci. Rep.* **10**, 11063 (2020). 32632136 10.1038/s41598-020-67528-y PMC7338421

[5] Grant, C. A. et al. Crop yield and nitrogen concentration with controlled release Urea and split applications of nitrogen as compared to non-coated Urea applied at seeding. *Field Crops Res.* **127**, 170–180 (2012).

[6] Zhang, X. et al. Managing nitrogen for sustainable development. *Nature* **528**, 51 (2015). 26595273 10.1038/nature15743

[7] Maqsood, M. A., Awan, K. U., Aziz, T., Ashraf, N. M. & Ali, M. Nitrogen management in calcareous soils: problems and solutions. *Pak. J. Agric. Sci.* **53**, 79–95 (2016).

[8] Wei, K. et al. Optimizing nitrogen and phosphorus application to improve soil organic carbon and alfalfa hay yield in alfalfa fields. *Front. Plant. Sci.* **14**, 1276580 (2024). 38312359 10.3389/fpls.2023.1276580 PMC10835404

[9] Rose, M. T. et al. & Patti A.F. A slow release nitrogen fertiliser produced by simultaneous granulation and superheated steam drying of Urea with brown coal. *Chem. Biol. Technol. Agric.* **3** (2016).

[10] Bakshi, S., Banik, C., Laird, D. A., Smith, R. & Brown, R. C. Enhancing Biochar as scaffolding for slow release of nitrogen fertilizer. *ACS Sustain. Chem. Eng.* **9**, 8222–8231 (2021).

[11] Kammann, C. I. et al. Plant growth improvement mediated by nitrate capture in co-composted Biochar. *Sci. Rep.* **5**, 11080 (2015). 26057083 10.1038/srep11080 PMC4460888

[12] Melo, L. C. A., Lehmann, J., Carneiro, J. S. S. & Camps-Arbestain, M. Biochar-based fertilizer effects on crop productivity: A meta-analysis. *Plant. Soil.* **472**, 45–58 (2022).

[13] Ahmed, N. et al. Effect of acidified Biochar on soil phosphorus availability and fertilizer use efficiency of maize (Zea mays L). *J. King Saud Univ. Sci.* **33**, 101635 (2021).

[14] Bhatnagar, A. & Sillanpää, M. A review of emerging adsorbents for nitrate removal from water. *Chem. Eng. J.* **168**, 493–504 (2011).

[15] Bashir, S. et al. Efficiency of KOH-modified rice straw-derived Biochar for reducing cadmium mobility, bioaccessibility and bioavailability risk index in red soil. *Pedospher* **30**, 874–882 (2020).

[16] Akanji, M. A., Ahmad, M., Al-Wabel, M. I. & Al-Farraj A.S.F. Soil phosphorus fractionation and bio-availability in a calcareous soil as affected by conocarpus waste Biochar and its acidified derivative. *Agric* **12**, 2157 (2022).

[17] Sahin, O. et al. Effect of acid modification of Biochar on nutrient availability and maize growth in a calcareous soil. *Soil. Use Manag*. **33**, 447–456 (2017).

[18] Guan, Y., Song, C., Gan, Y. & Li, F. M. Increased maize yield using slow-release attapulgite-coated fertilizers. *Agron. Sustain. Dev.* **34**, 657–665 (2014).

[19] Manzoor, S. et al. Biochar and slow-releasing nitrogen fertilizers improved growth, nitrogen use, yield, and fiber quality of cotton under arid climatic conditions. *Environ. Sci. Pollut. Res.* **29**, 13742–13755 (2022). 10.1007/s11356-021-16576-6 PMC8803770 34595718

[20] Zuluaga, D. L. & Sonnante, G. The use of nitrogen and its regulation in cereals: structural genes, transcription factors, and the role of MiRNAs. *Plants* **8**, 294 (2019). 31434274 10.3390/plants8080294 PMC6724420

[21] Rashid, M. et al. Carbon-based slow-release fertilizers for efficient nutrient management: synthesis, applications, and future research needs. *J. Soil. Sci. Plant. Nutr.* **21**, 1144–1169 (2021).

[22] Reconnaissance Soil Survey Campbellpur. Soil Survey of Pakistan (1970).

[23] Tandon, H. L. S. & Tiwari, K. N. *Methods of Analyses of Soils, Plants, Waters, Fertilizers, and Organic Manures* (Fertilizer Development and Consultation Organization New Delhi, 2009).

[24] Richards, L. A. *Diagnosis and Improvement of Saline and Alkali Soils* (US Government Printing Office, 1954).

[25] Nelson, D. .S. methods of soil analysis part 3. in chemical methods. *Soil. Sci. Soc. Am. Book. Series* 961–1010 (1996).

[26] Chen, M. J., Hsieh, Y. T., Weng, Y. M. & Chiou, R. Y. Y. Flame photometric determination of salinity in processed foods. *Food Chem.* **91**(4), 765–770 (2005).

[27] Sangakkara, R., Attanayake, I. K. B. & Stamp, P. Impact of locally derived organic materials and method of addition on maize yields and nitrogen use efficiencies in major and minor seasons of tropical South Asia. *Commun. Soil. Sci. Plant. Anal.* **39**, 2584–2596 (2008).

[28] da Silva, C. J. S. et al. A. Long-term effect of biochar-based fertilizers application in tropical soil: agronomic efficiency and phosphorus availability. *Sci. Tot Environ.* **760**, 143955 (2021). 10.1016/j.scitotenv.2020.143955 33341614

[29] Gwenzi, W., Nyambishi, T. J., Chaukura, N. & Mapope, N. Synthesis and nutrient release patterns of a biochar-based N-P-K slow-release fertilizer. *Internat J. Environ. Sci. Technol.* **15**, 405–414 (2018).

[30] Swami, K. et al. Starch wall of Urea: facile starch modification to residue-free stable Urea coating for sustained release and crop productivity. *Carbohydr. Polym.* **317**, 121042 (2023). 37364943 10.1016/j.carbpol.2023.121042

[31] Oladele, S. O. Changes in physcochemical properties and quality index of an Alfisol after three years of RHB amendment in rainfed rice – Maize cropping sequence. *Geoderma* **353**, 359–371 (2019).

[32] Hagemann, N. et al. Organic coating on Biochar explains its nutrient retention and stimulation of soil fertility. *Nat. Commun.* **8**, 1089 (2017). 29057875 10.1038/s41467-017-01123-0 PMC5715018

[33] Wang, C. et al. Biochar-based slow-release of fertilizers for sustainable agriculture: A mini-review. *Environ. Sci. Ecotechnol*. **10**, 100167 (2022). 36159737 10.1016/j.ese.2022.100167 PMC9488105

[34] Jeffery, S. et al. Biochar boosts tropical but not temperate crop yields. *Environ. Res. Lett.* **12**, 053001 (2017).

[35] Murtaza, G. et al. Recent trends and economic significance of modified/functionalized biochars for remediation of environmental pollutants. *Sci. Rep.* **14**(1), 217 (2024). 38167973 10.1038/s41598-023-50623-1 PMC10762257

[36] Murtaza, G. et al. I. A review of mechanism and adsorption capacities of biochar-based engineered composites for removing aquatic pollutants from contaminated water. *Front. Environ. Sci.* **10**, 1035865 (2022).

[37] Sahrawat, K. L. Redox potential and pH as major drivers of fertility in submerged rice soils: A conceptual framework for management. *Commun. Soil. Sci. Plant. Anal.* **46**, 1597–1606 (2015).

[38] Adeleke, R., Nwangburuka, C. & Oboirien, B. Origins, roles and the fate of organic acids in soils: A review. *S. Afr. J. Bot.* **108**, 393–406 (2017).

[39] Baloch, H. et al. Moringa leaf extract enhances the growth and yield characteristics of buckwheat genotypes by modulating the biochemical and physiological activities. *Asian J. Agric. Biol.* (4), 2023328. 10.35495/ajab.2023.328 (2024).

[40] Lentz, R. D. & Ippolito, J. A. Biochar and manure affect calcareous soil and corn silage nutrient concentrations and uptake. *J. Environ. Qual.* **41**, 1033–1043 (2012). 22751045 10.2134/jeq2011.0126

[41] Kharel, G. et al. Biochar surface oxygenation by ozonization for super high cation exchange capacity. *ACS Sus Chem. Eng.* **7** (19), 16410–16418 (2019).

[42] Huff, M. D., Marshall, S., Saeed, H. A. & Lee, J. W. Surface oxygenation of Biochar through ozonization for dramatically enhancing cation exchange capacity. *Bioresour. Bioprocess.* **5**, 18 (2018).

[43] Palansooriya, K. N. et al. Response of microbial communities to biochar-amended soils: A critical review. *Biochar* **1**, 3–22 (2019).

[44] Saranya, K., Kumutha, K. & Krishnan, P. S. Influence of Biochar and Azospirillum application on the growth of maize. *Madras Agric. J.* **98**, 58–164 (2011). (2011).

[45] Blanco-Canqui, H. Does Biochar improve all soil ecosystem services? *GBC Bioener*. **13**, 291–304 (2020).

[46] Li, M. et al. Responses of ammonia-oxidizing microorganisms to Biochar and compost amendments of heavy metals-polluted soil. *J. Environ. Sci.* **102**, 263–272 (2021). 10.1016/j.jes.2020.09.029 33637252

[47] Victoria, O. et al. Seed treatment with 24-epibrassinolide improves wheat germination under salinity stress. *Asian J. Agric. Biol.* **2023**(3): 2022076. 10.35495/ajab.2022.076

[48] Dong, D. et al. An effective biochar-based slow-release fertilizer for reducing nitrogen loss in paddy fields. *J. Soils Sediments*. **20**, 3027–3040 (2020).

[49] Gao, Y. et al. A critical review of biochar-based nitrogen fertilizers and their effects on crop production and the environment. *Biochar* **4**, 36 (2022).

[50] Jia, Y. M., Hu, Z. Y., Ba, Y. X. & Qi, W. F. Application of biochar-coated Urea controlled loss of fertilizer nitrogen and increased nitrogen use efficiency. *Chem. Biol. Technol. Agric.* **8**, 3 (2021).

[51] Cen, Z., Wei, L., Muthukumarappan, K., Sobhan, A. & Mcdaniel, R. Assessment of a biochar-based controlled release nitrogen fertilizer coated with polylactic acid. *J. Soil. Sci. Plant. Nut*. **21**, 2007–2019 (2021).

[52] Osei, A. F., Jin, X., Abdullah, W. Z. B. W. & Sidique, S. N. M. Silicon improves strawberry plants nutrient uptake and epicuticular wax formation in a rhizosphere cooling system. *Asian J. Agric. Biol.* **2023**(2): 2022060. 10.35495/ajab.2022.060 (2023).

[53] Vogeler, I., Blard, A. & Bolan, N. Modelling DCD effect on nitrate leaching under controlled conditions. *Soil. Res.* **45**, 310–317 (2007).

[54] Xiaojing, Y. et al. Combined effects of straw-derived Biochar and bio-based polymer-coated Urea on nitrogen use efficiency and cotton yield. Chem. *Speciat. Bioavail*. **30**, 112–122 (2018).

[55] Wang, H. H., Zheng-Yi, H. U. & Zhu, X. Q. Comparison of nitrogen loss after Biochar coated Urea and common Urea fertilization in vegetable soil at Chaihe catchment of dianchi lake. *J. Anhui Agric. Sci.* **43**, 104–107 (2015).

[56] Fatemi, R., Yarnia, M., Mohammadi, S., Vand, E. K. & Mirashkari, B. Screening barley genotypes in terms of some quantitative and qualitative characteristics under normal and water deficit stress conditions. *Asian J. Agric. Biol.* **2023**(2): 2022071. 10.35495/ajab.2022.071

[57] Khan, Z. et al. Coupling of Biochar with nitrogen supplements improve soil fertility, nitrogen utilization efficiency and rapeseed growth. *Agron* **10**, 1661 (2020).

[58] Laird, D., Fleming, P., Wang, B., Horton, R. & Karlen, D. Biochar impact on nutrient leaching from a Midwestern agricultural soil. *Geoder* **158**, 436–442 (2010).

[59] Qayyum, M. F., Steffens, D., Reisenauer, H. P. & Schubert, S. Kinetics of carbon mineralization of biochars compared with wheat straw in three soils. *J. Environ. Qual.* **41**, 1210–1220 (2012). 22751064 10.2134/jeq2011.0058

[60] Sindesi, O. A., Ncube, B., Lewu, M. N., Mulidzi, A. R. & Lewu, F. B. Cabbage and Swiss Chard yield, irrigation requirement and soil chemical responses in zeolite-amended sandy soil. *Asian J. Agric. Biol.* **2023**(1): 202111387. 10.35495/ajab.2021.11.387

[61] Ladha, J. K., Pathak, H., Krupnik, T. J., Six, J. & van Kessel, C. Efficiency of fertilizer nitrogen in cereal production: retrospects and prospects. *Adv. Agron.* **87**, 85–156 (2005).

[62] Raun, W. R. et al. Improving nitrogen use efficiency in cereal grain production with optical sensing and variable rate application. *Agron. J.* **94**, 815–820 (2002).

[63] Pangaribuan, D. H., Widagdo, S., Hariri, A. M., Siregar, S. & Sardio, M. I. The effect of rice straw mulch and cow urine on growth, yield, quality on sweet corn and pest population density. *Asian J. Agric. Biol.* **2023**(1): 202103123. 10.35495/ajab.2021.03.123 (2023).

[64] Zhou, L. et al. Effect of Biochar application on the improvement of soil properties and buckwheat (Fagopyrum esculentum Moench) yield on two contrasting soil types in a Semi-Arid region of inner Mongolia. *Agron* **14**, 1137 (2024).

